# Distance-Dependent Evolution of Electronic States in Kagome-Honeycomb Lateral Heterostructures in FeSn

**DOI:** 10.1021/acsnano.3c11381

**Published:** 2024-03-15

**Authors:** Tuan Anh Pham, Seoung-Hun Kang, Yasemin Ozbek, Mina Yoon, Pengpeng Zhang

**Affiliations:** † Department of Physics and Astronomy, Michigan State University, East Lansing, Michigan 48824, United States; ‡ Materials Science and Technology Division, Oak Ridge National Laboratory, Oak Ridge, Tennessee 37831, United States

**Keywords:** FeSn, Kagome lattice, flat band, lateral heterointerfaces, molecular beam epitaxy (MBE), scanning tunneling microscopy/spectroscopy (STM/STS), first-principles density functional theory calculations (DFT)

## Abstract

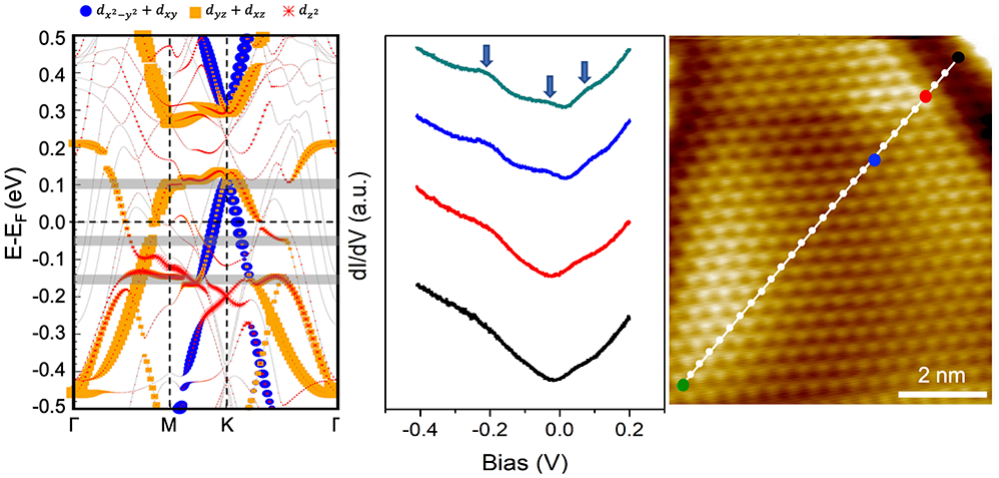

In this work, we demonstrate the formation and electronic influence of lateral heterointerfaces in FeSn containing Kagome and honeycomb layers. Lateral heterostructures offer spatially resolved property control, enabling the integration of dissimilar materials and promoting phenomena not typically observed in vertical heterostructures. Using the molecular beam epitaxy technique, we achieve a controllable synthesis of lateral heterostructures in the Kagome metal FeSn. With scanning tunneling microscopy/spectroscopy in conjunction with first-principles calculations, we provide a comprehensive understanding of the bonding motif connecting the Fe_3_Sn-terminated Kagome and Sn_2_-terminated honeycomb surfaces. More importantly, we reveal a distance-dependent evolution of the electronic states in the vicinity of the heterointerfaces. This evolution is significantly influenced by the orbital character of the flat bands. Our findings suggest an approach to modulate the electronic properties of the Kagome lattice, which should be beneficial for the development of future quantum devices.

An in-depth understanding of exotic quantum phases in condensed matter systems is highly imperative for the development and design of future quantum devices relying on strong electronic correlations. Among various condensed matter systems, Kagome lattices have been quickly emerging as one of the most important platforms for studying correlated and topological electronic states.^1^ In the Kagome lattice, atoms are arranged into a two-dimensional network comprising hexagons interspersed with triangles.^2^ Linearly dispersing Dirac band and nondispersing (flat) band are expected, with the latter arising from the destructive interference of Bloch wave functions that leads to the self-localization of electrons in the central honeycomb ring when only nearest-neighbor hopping is considered.^3,4^ To date, the Kagome lattice has been realized in a wide range of materials, including layered materials,^4−10^ non-van der Waals materials,^3,11^ and organometallic frameworks,^12−18^ which are expected to lead to the realization of correlated topological phases,^10,19,20^ excitonic Bose–Einstein condensation,^21,22^ spin liquid,^23,24^ unconventional charge density waves,^5,25−28^ magnetically intertwined superconductivity,^1,29,30^ and spintronic devices.^31^

Among the various types of Kagome lattice materials, the Kagome metal FeSn has attracted tremendous attention. Compared to its binary T_m_X_n_ sibling compounds (e.g., Fe_3_Sn_2_ and Fe_3_Sn), the Kagome planes in FeSn are spatially isolated from each other by the intercalation of Sn_2_ layers, making it the compound closest to the two-dimensional limit.^32−35^ Recent studies have revealed the presence of Dirac Fermions and flat bands in bulk FeSn crystals using angle-resolved photoemission spectroscopy and de Haas–van Alphen quantum oscillations.^35^ Theoretical studies have uncovered intricate details on the behavior of flat bands in Kagome metals.^36^ In particular, it has been shown that topological flat bands arise predominantly from the *d*_*z*2_ orbital, which is consistent with the symmetries of the Kagome lattice, including its 3-fold rotational symmetry. Strong crystal field splitting is essential to clearly distinguish these bands; in its absence, interorbital interactions can disrupt the stability of the flat band formation. Such findings contradict the widespread belief in the ubiquity of ideal flat bands in Kagome metals, because multiple *d*-orbitals with incompatible symmetries and/or insufficient crystal field splitting could prevent their manifestation.^36^ Nevertheless, the potential for strong electron correlation effects remains due to the high localization of the *d*-bands. Complementary to the bulk studies, epitaxial FeSn films grown on a substrate, SrTiO_3_ (STO) (111), were shown to exhibit surface flat bands that can potentially be integrated into heterostructures for various device applications.^37^ Previous investigations have primarily focused on vertical heterointerfaces along the stacking direction. A critical gap remains in the control of the lateral heterointerfaces formed between the Fe_3_Sn Kagome and Sn_2_ honeycomb layers and the thorough understanding of their influences on the electronic structures of the Kagome lattice. The study of the lateral heterointerfaces has proven challenging due to the absence of exposed Fe_3_Sn- and Sn_2_-terminated surfaces on the same plane in FeSn single crystals, which has limited the current understanding.

In this study, we report the formation and profound electronic implications of lateral heterostructures of Fe_3_Sn/Sn_2_ in FeSn thin films. The films are grown epitaxially on STO (111) using molecular beam epitaxy and investigated via the combination of scanning tunneling microscopy/spectroscopy (STM/STS) and density functional theory (DFT) calculations, which provide a comprehensive approach to understanding. The epitaxial growth of FeSn films on STO allows a thorough examination of the lateral Fe_3_Sn/Sn_2_ heterointerfaces, which is not possible in bulk FeSn single crystals. Our STS results reveal three distinct density of states peaks on the Fe_3_Sn-terminated surface. Those located at about −0.2 and 0.1 eV can be assigned to the surface flat bands of the Kagome lattice with 3*d* orbital character, as confirmed by the DFT calculations, whereas the peak at −0.05 eV may originate from the parabolic band by gaping out of a dispersive band of FeSn. In addition, we determined the bonding motif between the Fe_3_Sn- and Sn_2_-terminated surfaces at the lateral heterointerfaces. More notably, we discover an unusual long-range effect of the lateral heterointerface, where the surface flat bands are suppressed near the interface but recovered at a distance that depends on the orbital character of the state, as confirmed by STS line spectroscopy and DFT calculations. Our findings elucidate the impact of lateral heterointerfaces on the electronic behavior of the Kagome lattice, potentially providing a tool for engineering the electronic properties of FeSn to facilitate the development of future electronic and spintronic devices.

## Results and Discussions

**Figure 1:**
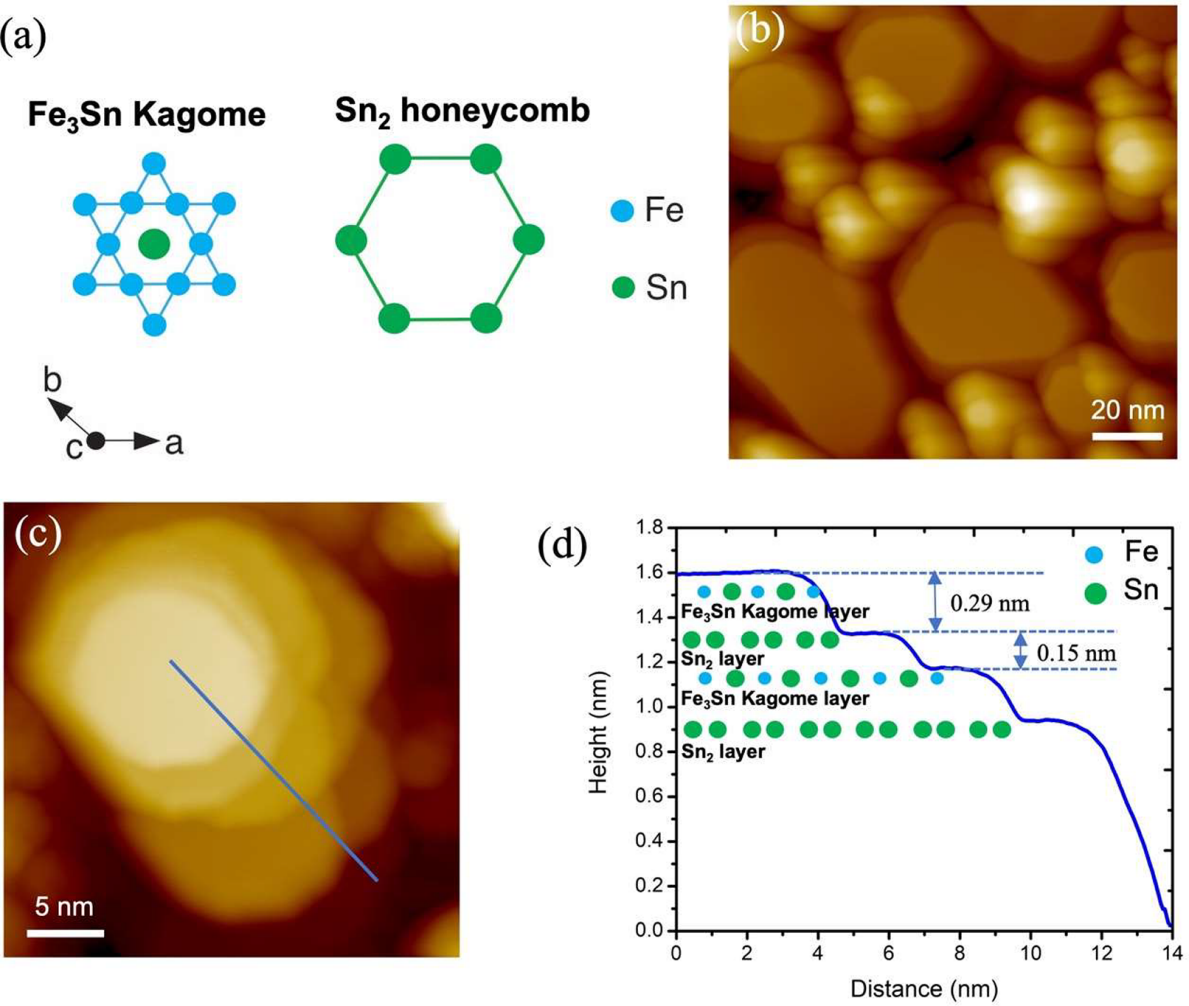
(a) In-plane schematics of the Fe_3_Sn Kagome and Sn_2_ honeycomb-terminated surfaces in FeSn. (b) Overview STM image (*V*_s_ = 4 V, *I*_t_ = 5 pA) showing the morphology of top-flat islands of FeSn epitaxially grown on STO (111) that follows the Volmer–Weber growth mode. (c) Close-up STM image (*V*_s_ = 3 V, *I*_t_ = 5 pA) illustrating the stacking of different layers in an isolated FeSn island. (d) Height profile taken along the blue line in (c), highlighting the unequal distances between the layers. The cartoon in (d) illustrates the alternating stacking of Fe_3_Sn Kagome and Sn_2_ layers along the *c*-axis of the FeSn island.

It was previously reported that the Kagome metal FeSn is a bulk antiferromagnet with Neel temperature *T*_N_ of 370 K.^38^ FeSn consists of an alternating stack of two-dimensional Fe_3_Sn Kagome layers and Sn_2_ honeycomb layers, as illustrated in Figure 1a, along the *c*-axis. Here, Fe spin moments within each Kagome layer are ferromagnetically aligned but antiferromagnetically coupled between adjacent layers.^39,40^ By tuning the growth parameters in the molecular beam epitaxy process, we are able to grow epitaxial films of FeSn on STO (111) with island sizes ranging from 20 to 70 nm. The growth of FeSn on STO (111) follows the Volmer–Weber mode, which is characterized by the formation of isolated three-dimensional flat-top islands, as shown in Figure 1b (see SI, Figure S1 and S2 for details). This result is in agreement with a recent study of the growth of FeSn films on STO (111) using the MBE technique.^41^ It should be noted that the distance between the two adjacent Kagome layers in the FeSn single crystal is measured to be ∼4.4 Å. However, as shown in the STM image and the corresponding line profile in Figure 1c–d, the distance between the Kagome topmost layer and the Sn_2_ underneath layer is about 2.9 Å, while that between the Sn_2_ layer and the second Kagome layer is approximately 1.5 Å. This result is consistent with the previous reports in bulk FeSn, which supports the stacking structure of the as grown thin films.^42,43^

**Figure 2:**
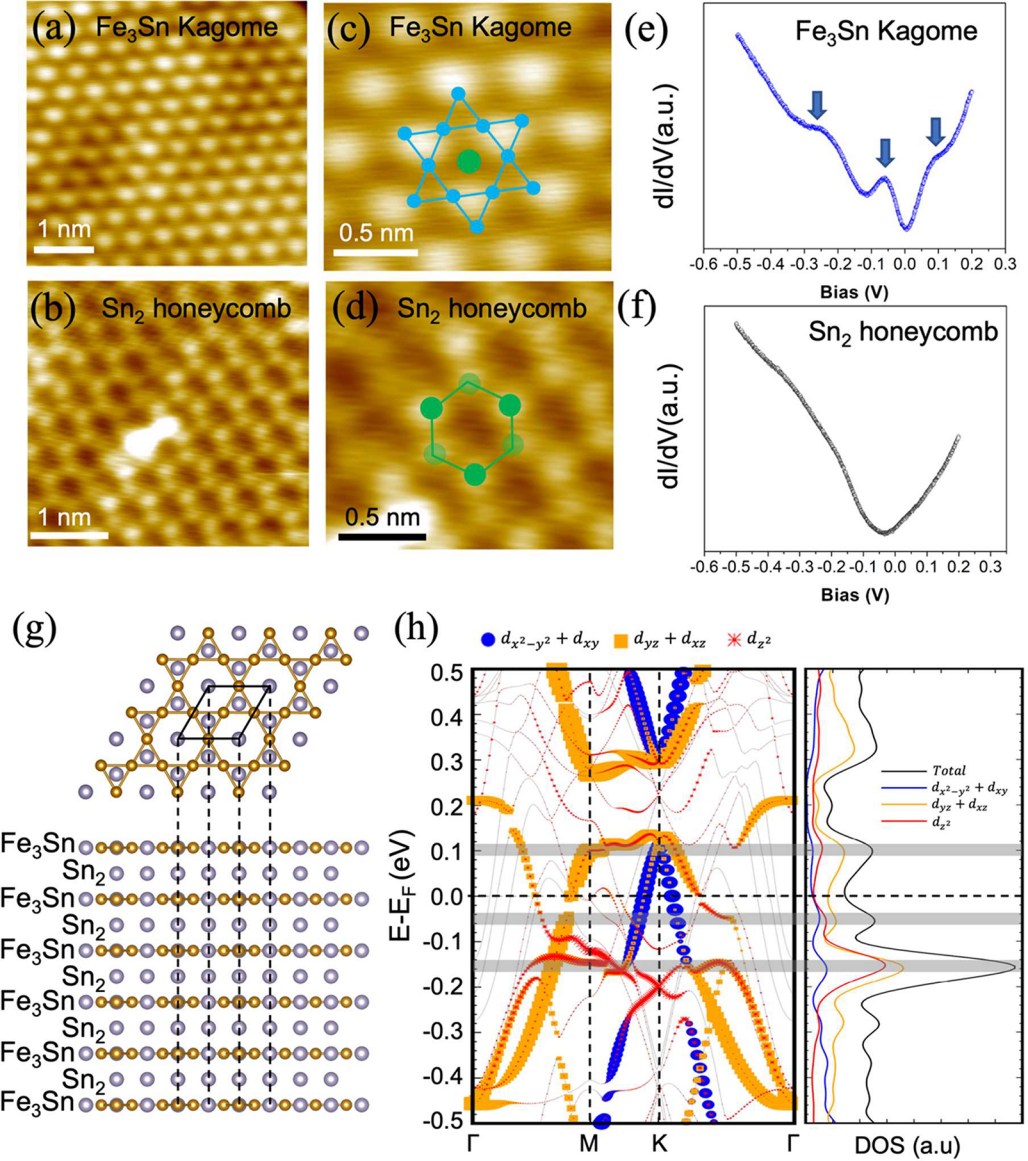
(a) A close-up STM image (*V*_s_ = −0.05 V, *I*_t_ = 600 pA) showing hexagonally close-packed bright protrusions of the Fe_3_Sn-terminated Kagome surface. (b) A close-up STM image (*V*_s_ = −0.05 V, *I*_t_ = 500 pA) showing the buckled honeycomb pattern of the Sn_2_-terminated surface. (c,d) Zoomed-in STM images (*V*_s_ = −0.05 V, *I*_t_ = 600 pA; *V*_s_ = −0.05 V, *I*_t_ = 500 pA) with the schematics overlaid to highlight the Star-of-David pattern of the Kagome surface and the buckled honeycomb pattern with three bright and three dark protrusions of the Sn_2_-terminated surface, respectively. Blue dots represent Fe atom, and green dots for Sn. (e,f) d*I*/d*V* spectra (set point: *V*_s_ = 0.3 V, *I*_t_ = 260 pA) taken on the Kagome and Sn_2_ layers, respectively. Three pronounced peaks are observed on the Kagome layer, as marked by the arrows in (e), while no significant peak is observed on the Sn_2_ layer. (g) Top and side views of the model structure of a 6-layer (6-Fe_3_Sn and 6-Sn_2_) slab. (h) Orbital projected band structure and density of states (DOS) calculated by DFT. The highlighted gray regions represent the energies at which the peaks occur, corresponding to the arrows in (e).

Parts a and b of Figure 2 show atomic resolution STM images of the Kagome- and Sn_2_-terminated surfaces of the FeSn film grown on STO (111), respectively. The Sn_2_-terminated surface shows a honeycomb pattern with three bright and three dark protrusions (illustrated by the green spheres of different shades in the schematics superimposed in Figure 2d), representing a buckled honeycomb lattice of Sn. In contrast, the Fe_3_Sn Kagome-terminated surface exhibits hexagonally close-packed bright protrusions,^32^ where the Kagome unit can be viewed as a Star-of-David pattern with a Sn atom located at the center,^32,43^ as illustrated in Figure 2c. To examine the electronic structures of the Kagome- and Sn_2_-terminated surfaces, we perform the differential conductance spectra shown in Figures 2e,f, respectively. Three pronounced peaks are observed in the d*I*/d*V* spectrum of the Fe_3_Sn Kagome surface: the two broad peaks are located at about −0.2 and 0.1 eV, while the narrow peak is located at ∼−0.05 eV. In contrast, no distinct features are found in the d*I*/d*V* spectrum of the Sn_2_ layer. In addition, our d*I*/d*V* maps taken simultaneously with the STM images show the characteristic Kagome pattern on the Fe_3_Sn layers in the bias range from −0.05 to −0.2 V (SI, Figure S3).

To gain a better understanding of the origin of the peaks in the d*I*/d*V* spectrum of the Kagome surface, we perform DFT calculations on a 6-layer FeSn slab terminated by the Kagome layer, as shown in Figure 2g. The analysis of the orbital characters of the DOS and the band structures (see Figure 2h) allow us to identify the characteristics of the high intensity peaks in the experimental d*I*/d*V* spectrum: the surface flat band of the Kagome lattice results in a broad peak near −0.2 eV, due to the contributions from both *d*_*z*2_ and (*d*_*xz*_ + *d*_*yz*_) orbitals of Fe atoms. The peak at 0.1 eV is attributed to the (*d*_*xz*_ + *d*_*yz*_) orbital character, which contributes to the surface flat band of the Kagome lattice along the M–K line. It is worth noting that there are two distinct types of flat bands in the Kagome layer of FeSn: the bulk flat band and the surface flat band.^35,37,43−45^ The bulk flat band is located at around 0.6 eV with the major contribution from the *d*_*xz*_/*d*_*yz*_ and *d*_*xy*_/*d*_*x*2–*y*2_ orbitals. Further details on the orbital characters of the electronic states of the Fe_3_Sn Kagome layer can be found in SI, Figure S4. The observation that bands having significant *d*_*z*2_ contributions are wide flat band within the Brillouin zone, contrasting with those from other *d* orbitals which form narrower flat bands, aligns closely with the latest theoretical findings.^36^ These findings emphasize the significance of *d*-orbital symmetry (from orbital rotation) and crystal field splitting in the formation of flat bands in the Kagome lattices. As for the peak located at −0.05 eV, it could result from a gap out by the hybridization between the nonorthogonal *d*_*xz*_/*d*_*yz*_ orbital of the Kagome surface and the bulk state (see SI, Figure S5 and the associated discussion). Although the interlayer coupling between the consecutive Kagome planes is suppressed by the Sn_2_ spacing layer in FeSn, hybridization between the Kagome layer and the adjacent Sn_2_ layer can be nonnegligible and impact the electronic structures of the Kagome lattice, which is similar to the role of intercalated layers in other two-dimensional materials.^46,47^

**Figure 3:**
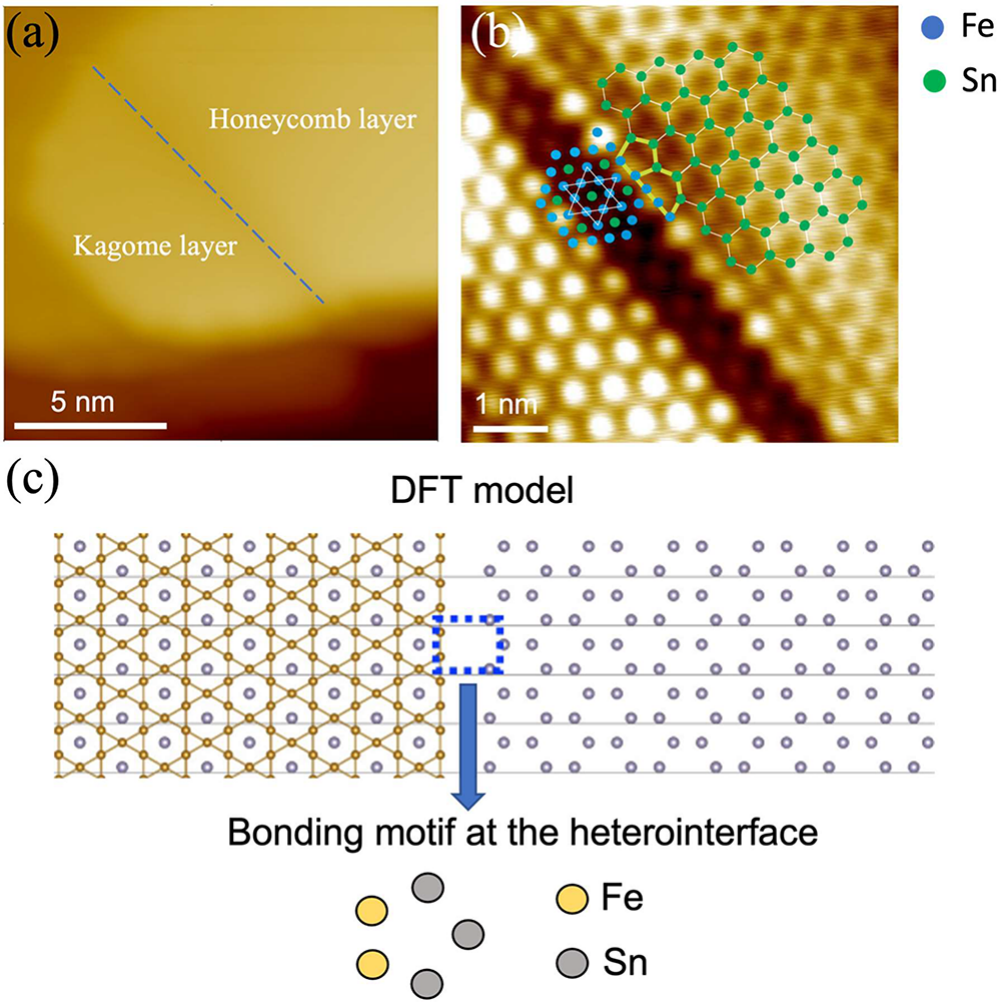
(a) STM image (*V*_s_ = 3 V, *I*_t_ = 5 pA) illustrating the in-plane boundary formed between the Fe_3_Sn Kagome and Sn_2_ honeycomb layers, as marked by the dashed blue line. (b) High-resolution STM image (*V*_s_ = 3 V, *I*_t_ = 5 pA) showing the atomic arrangement at the heterointerface boundary. The molecular models are overlaid on the STM image to illustrate how the Kagome and honeycomb layers are covalently linked together at the interface. (c) DFT model with the most energetically favorable bonding motif at the interface, which is consistent with that determined from the STM image in (b).

Interestingly, we observe lateral heterointerfaces formed between the Fe_3_Sn Kagome and Sn_2_-terminated surfaces (Figure 3a). Figure 3b shows a high-resolution STM image taken at the lateral heterointerface. The Kagome and honeycomb layers are identified on the lower left and upper right corners using the characteristics of the two surfaces discussed in Figure 2a–d. These two layers are on the same surface plane, as confirmed by the height profile analysis (SI, Figure S6), which could arise from the formation of stacking faults in the epitaxial film. The arrangement of individual Fe and Sn atoms at the interface is illustrated in the STM image. The honeycomb surface displays a zigzag edge, while the Kagome surface features a straight edge. The two different lattices are covalently linked by Fe–Sn bonds in the pentagon–heptagon pairs (5–7 rings) that consist of Fe and Sn atoms, which is considered the motif driving the formation of the heterointerface.

We further performed DFT calculations to gain insights into the chemical configuration of the bonding at the Kagome–honeycomb interface. Figure 3c shows the calculated molecular model of the boundary, which is energetically more favorable than the various other bonding possibilities, as illustrated in SI, Figure S7. Apparently, it fits very well with the pentagon–heptagon configuration determined from the STM images, which is responsible for the formation of the Kagome–honeycomb interface found in this work. From high-resolution STM images, we occasionally observe another type of bonding motif at the interface, as depicted in SI, Figure S8. The presence of this configuration may be related to some intermediate states during the formation of the interface.

**Figure 4:**
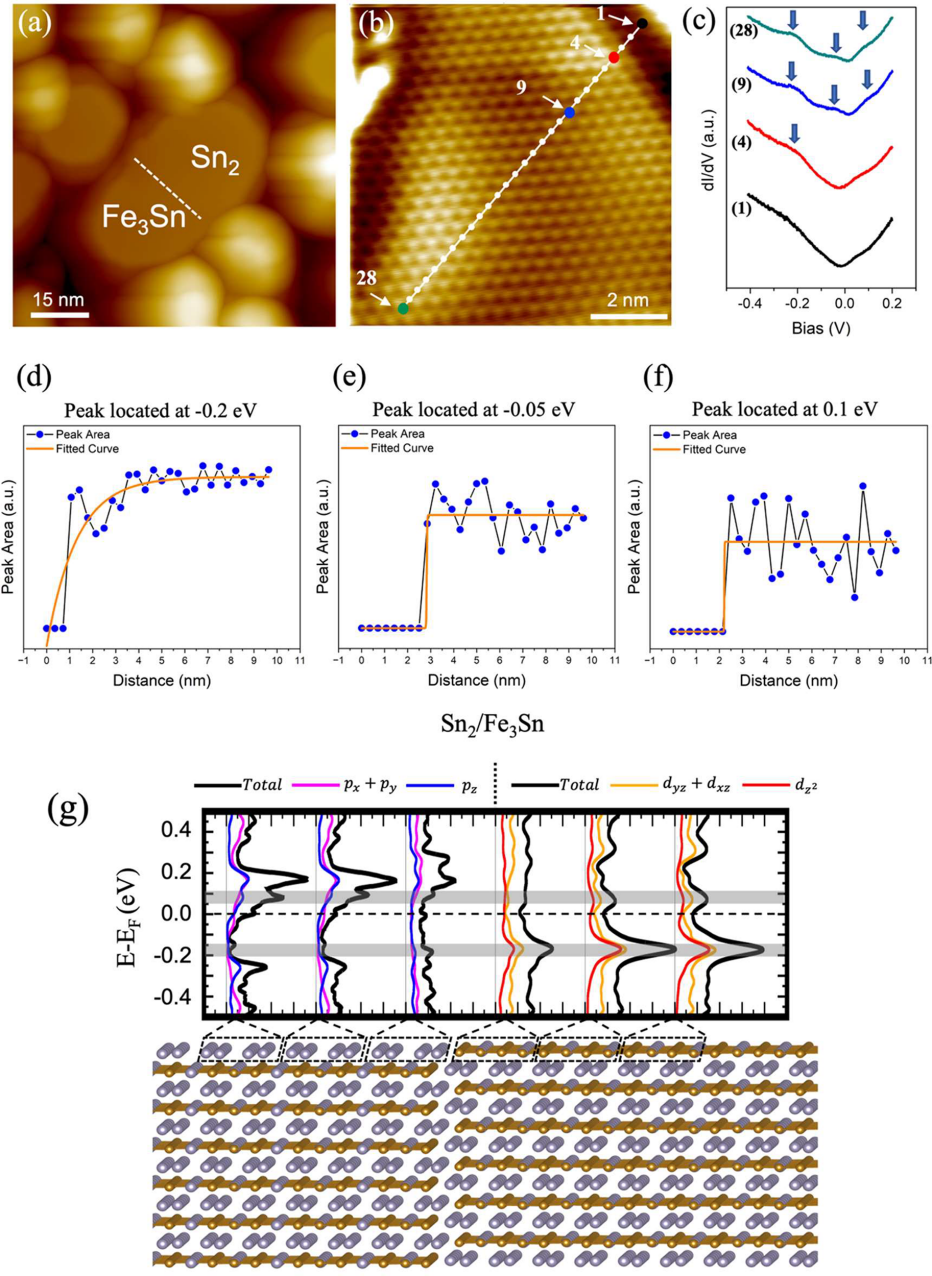
(a) Overview STM image (*V*_s_ = 3 V, *I*_t_ = 5 pA) illustrating the heterointerface boundary where the line STS spectra are taken. (b) Zoomed-in STM image (*V*_s_ = −0.05 V, *I*_t_ = 600 pA) into the Kagome surface area next to the heterointerface boundary. (c) The line STS spectra (set point: *V*_s_ = 0.4 V, *I*_t_ = 340 pA) are taken along the white trace from the heterointerface into the Kagome surface area shown in (b). (d–f) Areas of the peaks located at ∼−0.2, −0.05, and 0.1 eV vs distance from the heterointerface boundary extracted from the STS line spectra. Solid orange curves are the fittings. (g) Partial orbital projected density of states (PDOS) for the top layer atoms at the heterointerface boundary, based on the 6-layer FeSn slab structure. The highlighted gray regions represent the energies at which the peaks occur, corresponding to the arrows at −0.2 and 0.1 V in (c).

Furthermore, based on our line d*I*/d*V* spectroscopy measurements, we find that the lateral heterointerface significantly impacts the electronic structures of the Kagome lattice in areas close to the boundary. Figure 4a and SI, Figure S9b show an FeSn island and the corresponding Kagome–honeycomb lateral heterointerface with the bonding motif identical to that previously described in Figure 3. STS spectra taken across the boundary reveal that the top right area is a Sn_2_-terminated surface while the bottom left area is the Fe_3_Sn Kagome surface (see SI, Figure S9). Figure 4b depicts the trace of the STS spectra from a start point located right next to the boundary (point 1) to an end point located far away from the boundary on the Fe_3_Sn Kagome surface (point 28). Apparently, the STS curves taken right next to the boundary do not show any characteristic peaks of the Fe_3_Sn Kagome layer (point 1 in Figure 4b and the corresponding black curve in Figure 4c), suggesting that the heterointerface boundary suppresses the electronic structure and surface flat bands of Fe_3_Sn. At locations far away from the boundary, the characteristic peaks of the Fe_3_Sn Kagome surface are recovered (point 28 in Figure 4b and the corresponding green curve in Figure 4c). Along the path, the peak located at about −0.2 eV appears first, as illustrated in the spectrum taken at point 4 (red curve in Figure 4c). In contrast, the peaks at ∼−0.05 and 0.1 eV do not emerge until further away from the boundary edge, as marked by the blue curve (point 9) in Figure 4c, and once established, these peaks remain nearly unchanged, i.e., from point 9 (blue dot in Figure 4b) to point 28 (green dot in Figure 4b).

To be quantitative on the distance-dependence of the peak evolution from the heterointerface boundary, we plot the areas of the three distinct peaks, as shown in Figure 4d–f, respectively, for the entire 28 STS line spectra and obtain the fitting curves. Detailed information on the analysis, as well as the fitting parameters, can be found in SI, Figures S10,S11. Our results unambiguously reveal long-range heterointerface effects on the electronic structures of the Fe_3_Sn lattice. The overall trends of the distance-dependent evolution are similar between the electronic states at ∼−0.05 and 0.1 eV, but different from that at ∼−0.2 eV, as evidenced by the different functions used in the fittings. The “coupling” length to the heterointerface is ∼1.3 nm for the peak at −0.2 eV, while for the peaks located at −0.05 and 0.1 eV, this length is about 2.8 and 2.2 nm, respectively. Note that STM images display the convolution of geometric and electronic structures. The suppression of the electronic states near the lateral heterointerface could lead to perturbations to the morphology, e.g., the dark regions next to the boundary on the Kagome layer (Figures 3b and 4b).

To elucidate the long-range effects of the lateral heterointerface, we performed DFT calculations. Our model system, consisting of a lateral heterointerface connecting Fe_3_Sn and Sn_2_ terminated surfaces (Figure 3c), is 6 layers thick (Figure 4g). Only a peak at ∼−0.2 eV is visible close to the lateral heterointerface, and its intensity increases as it is further away from the boundary. Conversely, the peak at 0.1 eV, another surface flat band feature as discussed earlier, is not visible near the boundary, appearing slightly away. This result agrees well with the distance dependence of the peak evolution observed in the experiments, which can be attributed to the orbital character of the electronic states. The peak at 0.1 eV contains the major contributions from the (*d*_*xz*_*+ d*_*yz*_) orbitals. As these states with the *xy*-plane component interact with the *p* orbitals of the Sn_2_-terminated surface at the same energy, its intensity near the lateral heterointerface disappears. In contrast, the peak at −0.2 eV is contributed to by the *d*_*z*2_ and (*d*_*xz*_ + *d*_*yz*_) orbitals together. The number of the *p* orbital states on the Sn_2_-terminated surface at the same energy level is very small compared to the number of the states at 0.1 eV, therefore the interaction with the *d*_*z*2_ and (*d*_*xz*_ + *d*_*yz*_) of Fe_3_Sn is rather weak, resulting in only a reduction instead of complete suppression of the peak at −0.2 eV near the lateral heterointerface. This observation clearly illustrates the orbital selectivity of the flat band in the lateral heterostructures. Lastly, the peak at −0.05 eV observed in the experiment is absent in the DFT results. As shown in Figure 4d–f, the three Fe_3_Sn states have different “coupling” lengths with the heterointerface. This length is longest at ∼3 nm for the peak at −0.05 eV. Unfortunately, the lateral distance between the bulk and the heterointerface is less than 3 nm in our atomic model due to the limitation of the number of atoms in the DFT. Therefore, the peak at −0.05 eV, with the most extended interaction length with the heterointerface observed in the experiment, is not captured in DFT.

In addition, we have also identified Fe_3_Sn–Fe_3_Sn and Sn_2_–Sn_2_ homointerfaces formed between two different Fe_3_Sn or Sn_2_ domains. Detailed information can be found in SI, Figures S13 and S14. These boundaries have no pronounced influence on the electronic structures of the adjacent domains, different from the case for the heterointerfaces. We attribute it to the formation of a “defect”-like boundary with phase slips at the homointerface as compared to the strong covalent bonding that leads to the influence of the Sn_2_ electronic states on that of Fe_3_Sn at the “sharp” heterointerface.

## Conclusions

In this work, we investigate the effects of the Kagome–honeycomb lateral heterointerface on the flat bands and electronic structures of the Fe_3_Sn Kagome layer via a combination of STM/STS experiments and DFT calculations. Lateral heterointerfaces are established in the FeSn thin films epitaxially grown on the STO (111) substrate by MBE. Our STS data show the distinct peaks on the Kagome surface, whose origin and orbital characters are thoroughly explained by the DFT calculations. We further demonstrate that the Kagome–honeycomb lateral heterointerface has profound and long-range impacts on the electronic structures of the Kagome layer. Particularly, the two surface flat bands of the Kagome layer with the different orbital characters respond differently to the Kagome–honeycomb interface. This study and the orbital selectivity mechanism give rise to the potential for engineering the electronic properties of Kagome metal FeSn to facilitate the development of future quantum devices.

## Experimental Method

The substrate preparation and sample growth were performed in a standard molecular beam epitaxy (MBE) chamber with a base pressure of 6 × 10^–10^ mbar. After treatment, samples were directly transferred *in situ* into an Omicron low temperature scanning tunneling microscope (STM) operated at the liquid nitrogen temperature (∼77.5K) with based pressure of 1.8 × 10^–11^ mbar for characterization. Before deposition, the Nb-doped (0.5% by weight) SrTiO_3_(111) was cleaned using acetone and 2-propanol and then immediately transferred to the MBE chamber. The substrate was slowly heated and kept at 400 °C for 60 min to ensure a complete degassing. The substrate was then annealed at approximately 1150 °C using direct current heating for 60 min and then gradually cooled down to obtain a proper smooth and clean surface for the growth. FeSn was deposited onto the substrate in the MBE chamber by codeposition of pure Sn and Fe using two different electron beam evaporators with flux current of 200 nA and 0.4 nA, respectively. The substrate was maintained at around 530 °C during growth using resistive heating facilities. The temperature of the substrate was monitored by a thermocouple mounted at the heating stage in the MBE chamber. STS spectra were obtained using a lock-in amplifier with the modulation signal set at 26 meV in amplitude and 1.1 kHz in frequency. The STM tip was calibrated by measuring reference spectra on the silver substrate to avoid tip artifacts.

### Theoretical Approaches

We performed *ab initio* calculations based on density functional theory (DFT)^48,49^ as implemented in the Vienna Ab Initio Simulation Package (VASP)^50,51^ with projector augmented wave potentials^52,53^ and spin polarization. The Perdew–Burke–Ernzerhof (PBE) form^54^ was employed for the exchange-correlation functional with the generalized gradient approximation (GGA). The energy cutoff was set to 500 eV for all of the calculations. The Brillouin zone was sampled with a 25 × 25 × 1 Γ-centered k-grid. Atomic relaxations were performed until the Helmann–Feynman force acting on each atom became smaller than 0.01 eV/Å. FeSn adopts the *P*6/*mmm* space group, with the unit cell containing two Kagome layers composed of Fe atoms. These layers are enclosed by the Sn layer along the out-of-plane direction. The lattice parameters of bulk FeSn are found to be *a* = *b* = 5.285 Å and *c* = 4.444 Å. These values are in agreement with experimental measurements of *a* = *b* = 5.298 Å and *c* = 4.448 Å.^55^

## Supporting Information Available

Growth morphology of FeSn thin films, STM and simultaneously obtained d*I*/d*V* maps obtained at different biases on the Kagome-terminated surface, orbital origin of the Kagome layer electronic structure by DFT, bonding motif of the Kagome-honeycomb lateral heterointerface, quantitative analysis of the STS curves, homointerfaces formed between the Kagome–Kagome or honeycomb–honeycomb lattices (PDF)
