# Supplementary material for: Distance-Dependent Evolution of Electronic States in Kagome-Honeycomb Lateral Heterostructures in FeSn
Source: ACS Nano. 2024 Mar 15;18(12):8768–76. doi: 10.1021/acsnano.3c11381 (PMC10976957; doi:10.1021/acsnano.3c11381)
Supplement: Supplementary file 1 — nn3c11381_si_001.pdf [file nn3c11381_si_001.pdf]

Distance-Dependent Evolution of Electronic States in Kagome-Honeycomb Lateral Heterostructures in FeSn

Tuan Anh Pham, [1,#] Seoung-Hun Kang, [2,#] Yasemin Ozbek, [1] Mina Yoon, [2,*] and Pengpeng Zhang[1,*]

[1]Department of Physics and Astronomy, Michigan State University, East Lansing, MI, 48824, U.S.A.

[2]Materials Science and Technology Division, Oak Ridge National Laboratory, Oak Ridge, TN, 37831, U.S.A.

Email: myoon@ornl.gov; zhangpe@msu.edu
# These authors contributed equally.

Section SI: Growth morphology of FeSn thin films

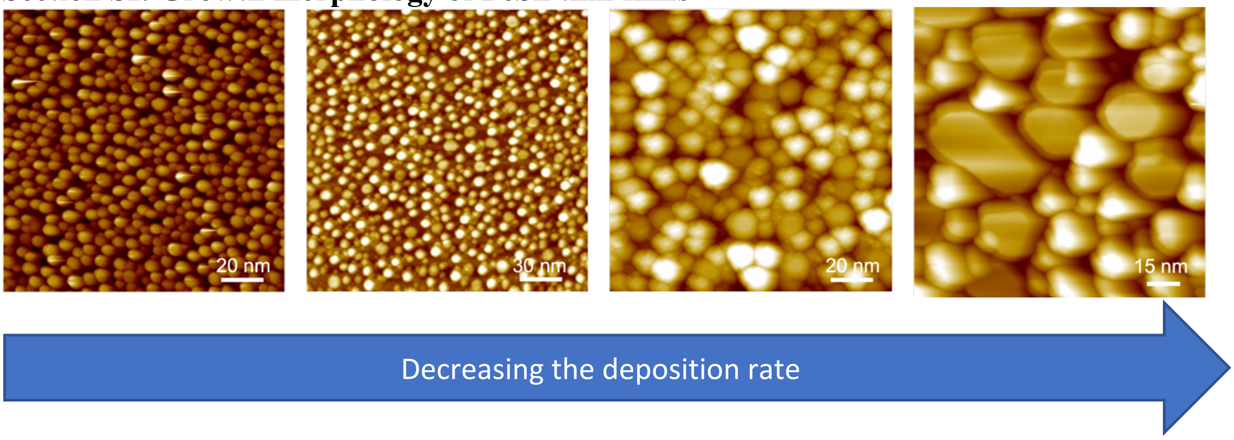

Figure S1. A series of STM images (V_s = 4 V, I_t = 5 pA) taken on the various FeSn samples. When the deposition rate is decreased, the FeSn island size gets larger from around 5 nm to 70 nm, revealing that the epitaxial growth of FeSn on STO can be kinetically limited.

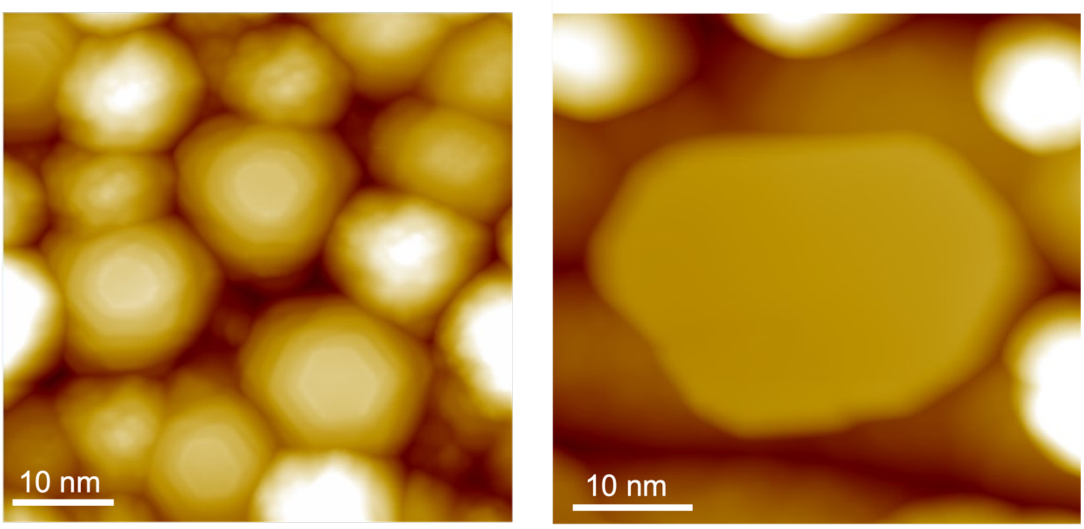

Figure S2. STM images (V_s = 4 V, I_t = 5 pA) showing the formation of isolated FeSn islands with flat top on STO(111), confirming that the epitaxial growth is characterized by the Volmer-Weber growth mode.

It is challenging to epitaxially grow FeSn films with a high degree of uniformity and sharp interfaces within the lateral heterostructures. In our study, Fe and Sn were deposited using electron-beam evaporators with the evaporation rate carefully controlled by the flux current. Growth was performed under Sn-rich conditions to optimize film quality. We started our process by exploring four primary growth parameters: Fe flux, Sn flux, substrate temperature, and deposition time. Each of these factors had two levels – for Sn flux, we used 150 nA (low) and 400 nA (high); for Fe flux, the levels were 0.2 nA (low) and 1 nA (high); substrate temperature ranged from 350 ^{O}C (low) to 580 ^{O}C (high); and deposition time varied between 2 hours (low) to 8 hours (high). In this initial phase, 16 experimental runs (2×2×2×2) were performed to analyze the results of each combination.

Our results indicated that the growth of FeSn is kinetically limited, and larger FeSn islands are formed at slower deposition rates as shown in **Figure S1**. These findings guided our subsequent growth where we fine-tuned the parameters to achieve heterostructures with a high degree of uniformity. The final growth parameters used, as mentioned in the experimental section of the manuscript, were 200 nA Sn flux, 0.4 nA Fe flux, substrate temperature of 530 ^{O}C, and deposition time of 6 hours. It should be noted that other factors such as substrate-source distances, vacuum conditions, temperature calibration and thermocouple position, etc., could also affect the growth performance.

Section SII: STM and simultaneously obtained *dI/dV* maps obtained at different biases on the Kagome-terminated surface

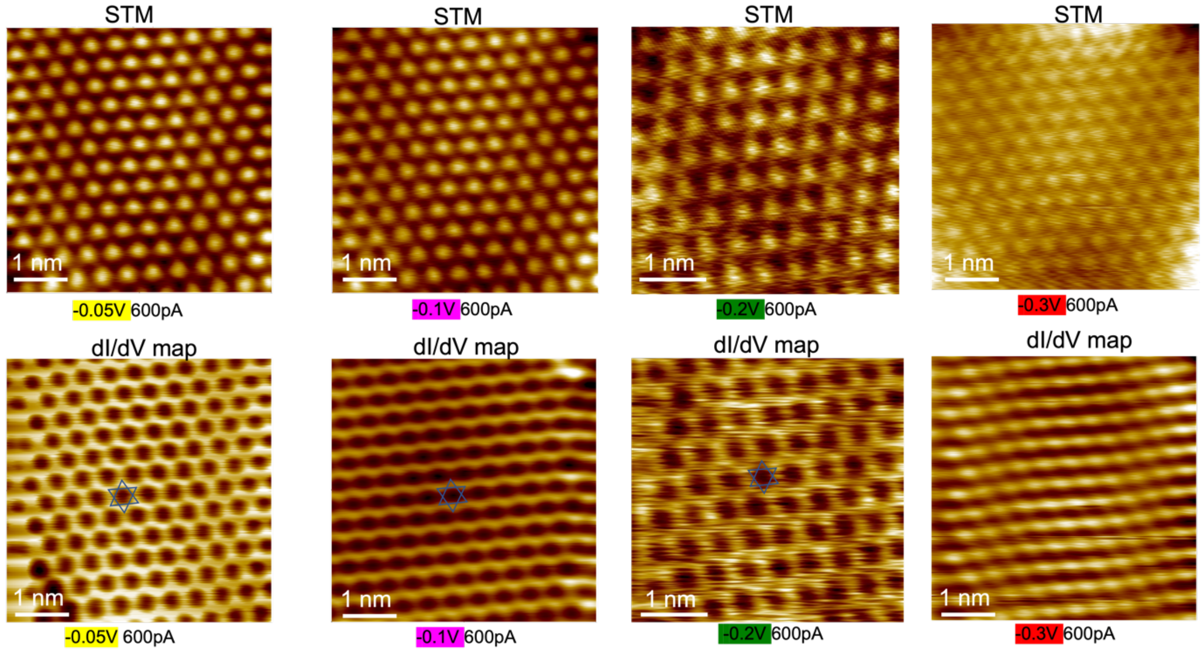

Figure S3. A series of STM images and the corresponding *dI/dV* maps showing Kagome patterns in the bias range of -0.05 V to -0.2 V. These results are consistent with our DFT calculations of the band structure which reveal the less dispersive bands at -0.05 V and -0.2 V.

Section SIII: Orbital origin of the Kagome layer electronic structure by DFT

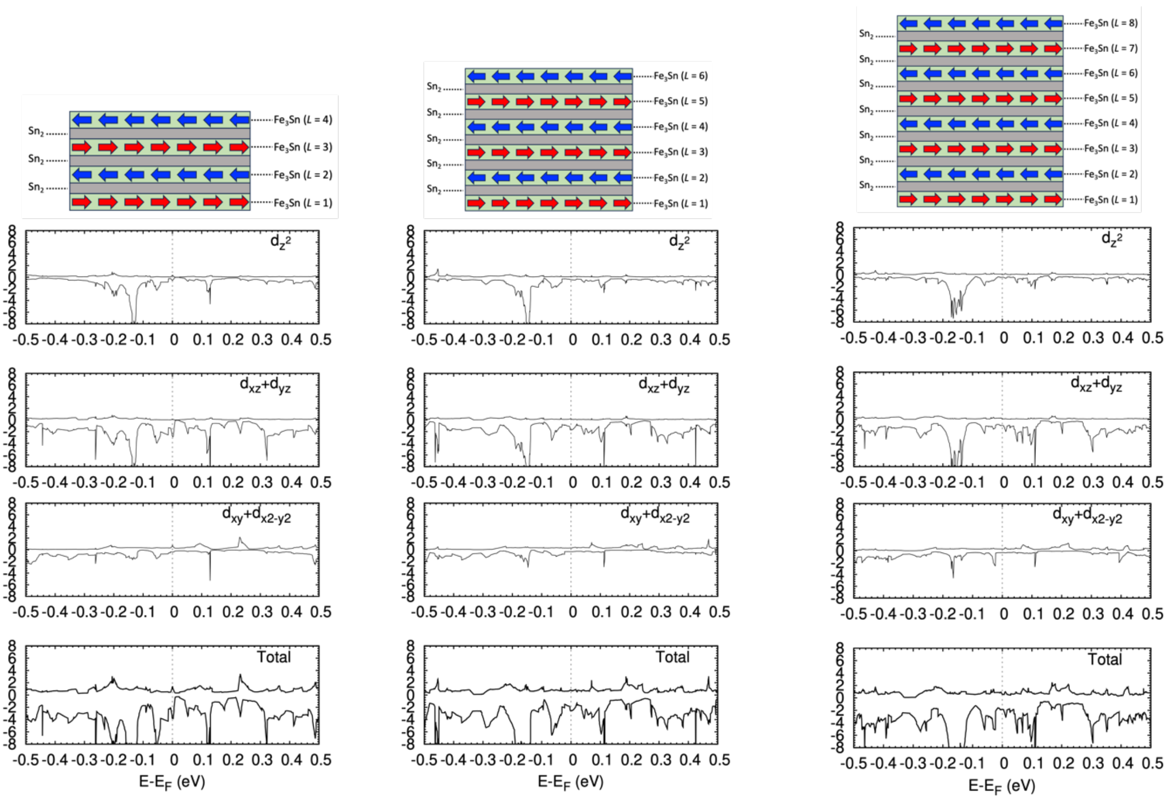

Figure S4. Orbital-projected density of states of the Kagome-terminated surface at different slab thickness. The calculated results clearly support that both d_{z2} and (d_{xz} + d_{yz}) orbitals contribute to the surface flat band at the bias close to -0.2 V, while the peak at 0.1 V is mainly attributed to the (d_{xz} + d_{yz}) orbital.

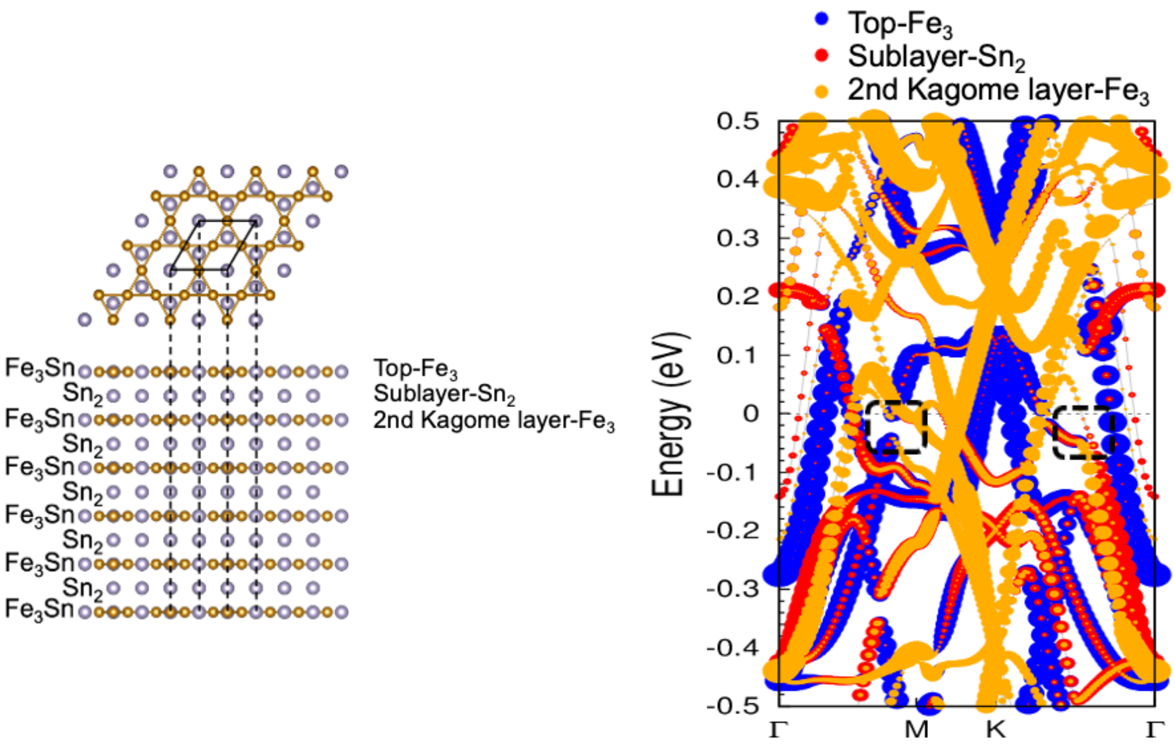

Figure S5. The projected electronic band structures of the FeSn slab for the topmost Kagome-Fe_3, sublayer Sn_2, and second Kagome-Fe_3. The blue, red, and orange colors represent the top Kagome-Fe_3, sublayer Sn_2, and second Kagome-Fe_3, respectively. Dotted square regions show the interaction of these layers.

For the binary Kagome compounds T_mX_n (T: Fe, Mn, Co; X: Sn, Ge), the stacking sequence of the Kagome layers varies with the m:n ratio. In FeSn (m:n=1:1), the Fe_3Sn Kagome plane and the spacing Sn_2 plane are stacked alternatively along the c axis, where the interlayer coupling *via* orbital hybridization or charge transfer between the neighboring Kagome planes is suppressed by the Sn_2 layer in the middle. In this regard, FeSn is the compound closest to the two-dimensional limit, as compared to Fe_3Sn_2 or Fe_3Sn. However, hybridization between the Kagome layer and the adjacent Sn_2 layer can be nonnegligible and impact the electronic structures of the Kagome lattice.

In **Figure S5**, the layer-by-layer projected bands show the clear separation between the states of the surface Kagome layer ("Top-Fe_3" in blue) and the subsurface Kagome layer ("2nd Kagome layer – Fe_3", in yellow), suggesting the reduced interlayer coupling between the consecutive Kagome planes. On the other hand, as noted in our study and shown in **Figure S5**, there is still notable hybridization between the Kagome and the adjacent Sn_2 layers, which affects the electronic structure. Within the highlighted region (black dotted squares), the upper

Kagome Fe_3 layer (in blue) combines with the lower Sn_2 layer (in red) to form a composite band with a small contribution from the Sn_2, indicating the orbital hybridization between these two layers. This hybridization, especially between the top Fe_3 and Sn_2 layers, is responsible for the bandgap formation when interacting with the second Kagome Fe_3 layer (in orange) below. These features are particularly pronounced at energies around -0.05 eV.

Section SIV: Bonding motif of the Kagome-honeycomb lateral heterointerface

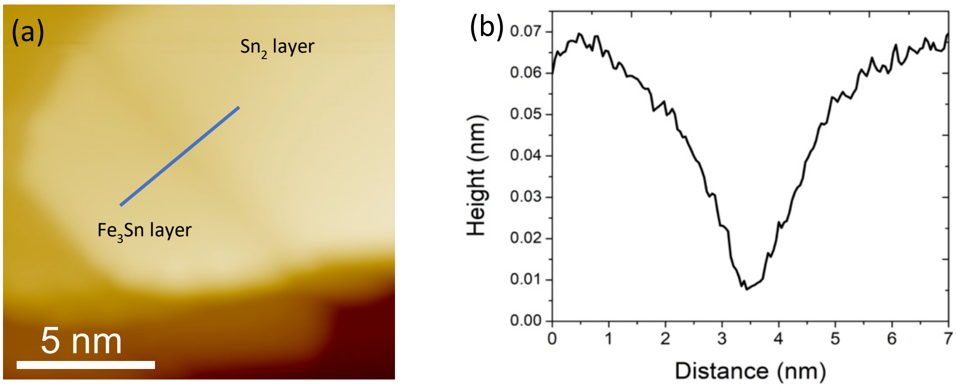

Figure S6. (a) STM image (V_s = 3 V, I_t = 5 pA) and (b) the corresponding height profile taken along the blue mark in (a), confirming that the Fe_3Sn and Sn_2 layers are on the same plane and that the interface formed between these layers is the lateral heterointerface.

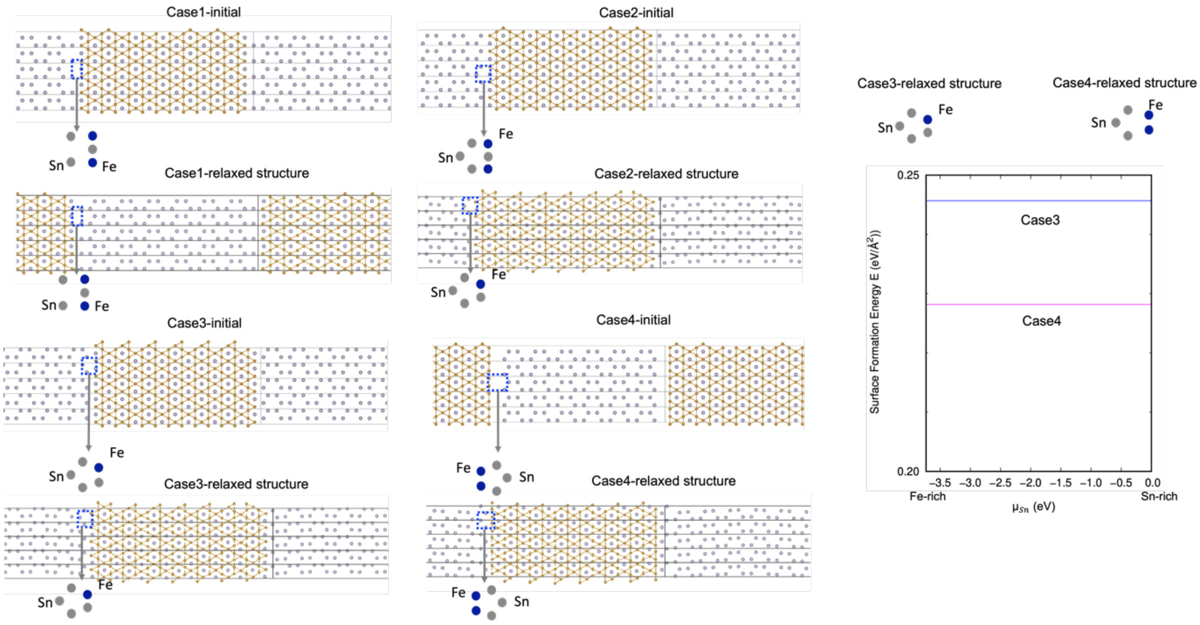

Figure S7. Formation energies of the different bonding motifs at the lateral Fe_3Sn/Sn_2 heterointerface calculated by DFT. The results reveal the pentagon-heptagon configuration

(case 4) as the most energetically favoured bonding motif at the covalently bonded lateral heterointerface. Case 1 configuration is much more unstable than the configurations of case 3 and 4.

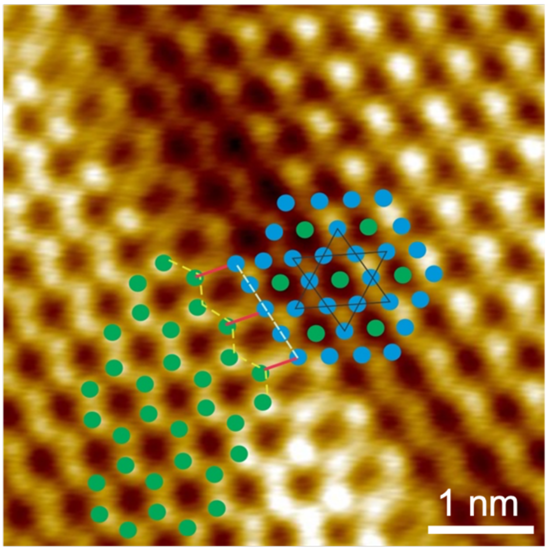

Figure S8. STM image (V_s = -0.05 V, I_t = 500 pA) suggesting another type of bonding motif at the Kagome-honeycomb heterointerface. This bonding motif is not revealed in the DFT calculations.

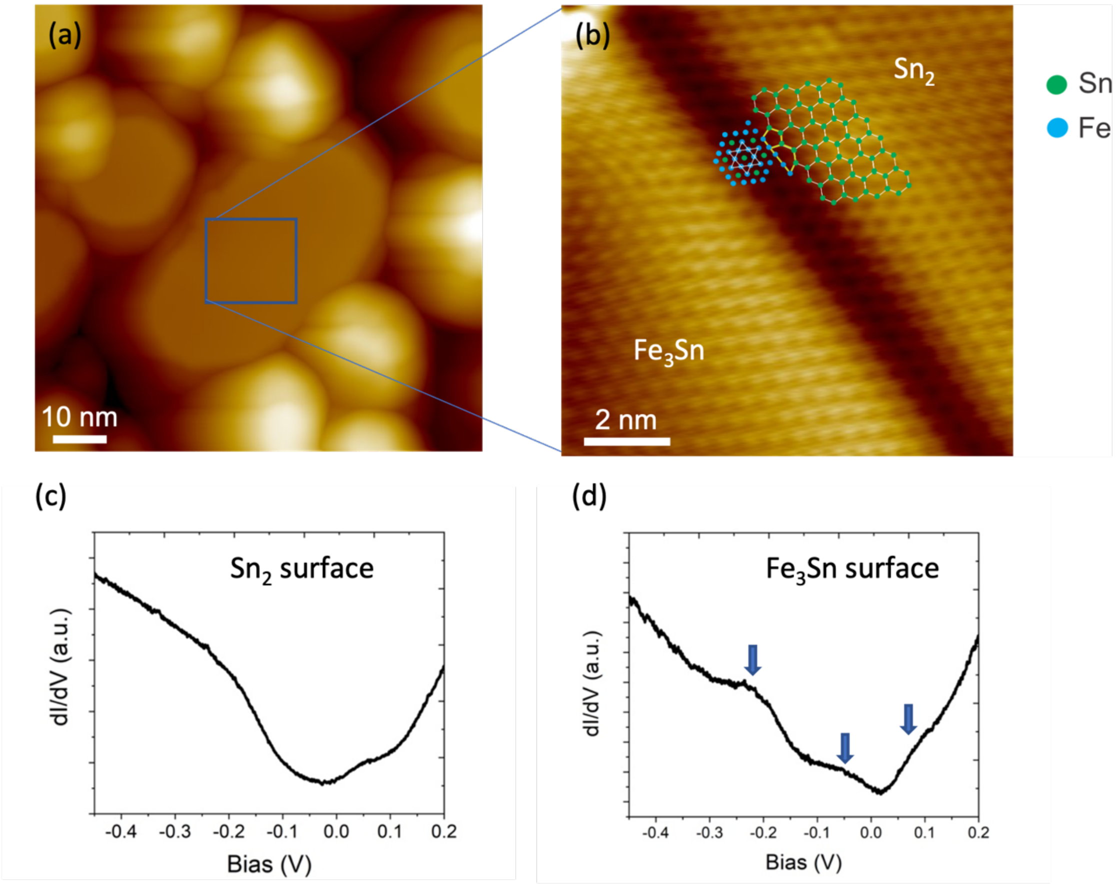

Figure S9. Overview STM image (V_s = 3 V, I_t = 5 pA) and zoomed-in STM image (V_s = -0.05 V, I_t = 600 pA) of the lateral heterointerface formed between the Fe_3Sn Kagome and the Sn_2 honeycomb layers that is shown in Figure 4 of the manuscript. The overlaid molecular models on the STM image in (b) highlights the bonding motif at the interface, which is consistent with that illustrated in Figure 3 in the manuscript. (c-d) The line STS spectra (setpoint: V_s = 0.4 V, I_t = 340 pA) confirm that the top right domain is terminated with the Sn_2 surface while the bottom left domain is terminated with the Kagome surface.

Section SV: Quantitative analysis of the STS curves

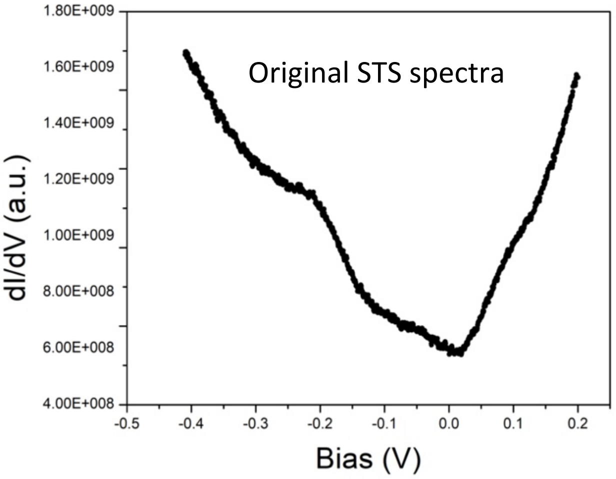

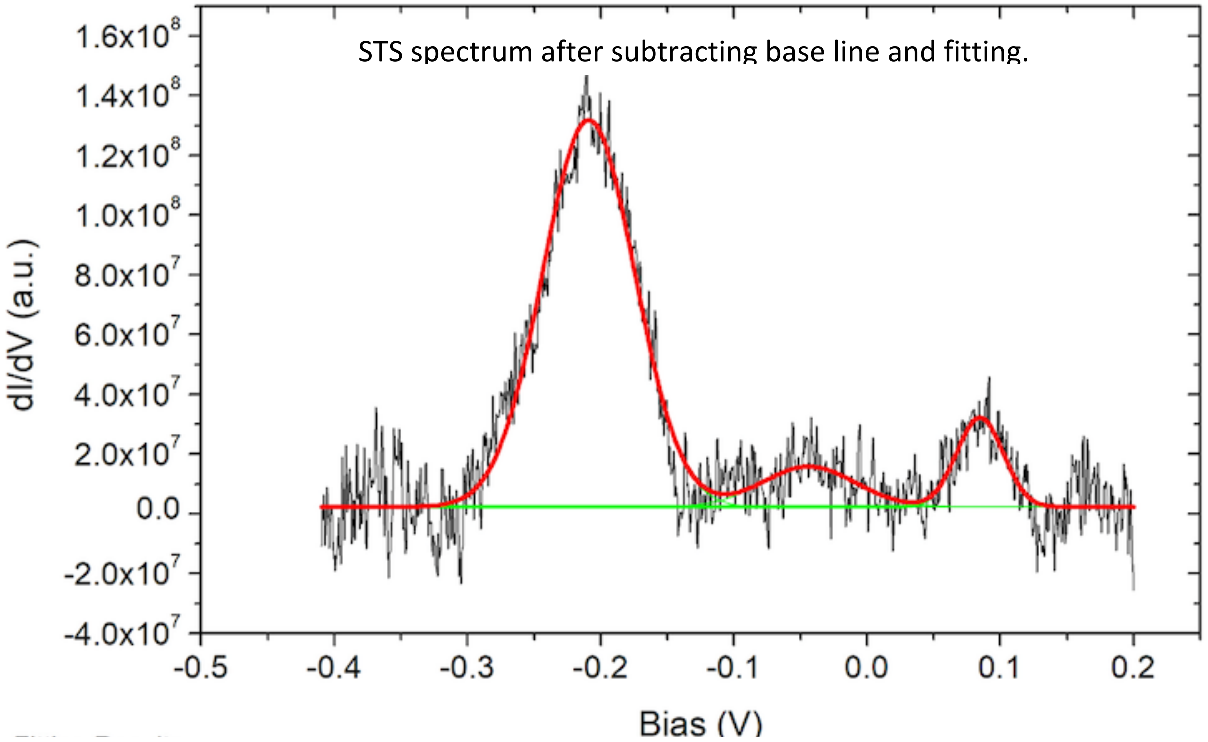

Fitting Results

Peak #	Peak Type	Area Intg (a.u.)	FWHM (V)	Max Height (a.u.)	Center Grvty (V)
1	Gaussian	12615270.17253	0.08133	129693822.47426	-0.20908
2	Gaussian	2575438.59314	0.08222	13576373.64966	-0.04373
3	Gaussian	2697577.83547	0.04105	29990664.97323	0.0846

Figure S10. The original STS spectrum (top) and the STS spectrum after subtracting the baseline (bottom). There are three characteristic peaks on the Kagome Fe_3Sn surface. Gaussian fitting of the peaks is shown in red. Area integration, full width at half maximum (FWHM), maximum height of each peak, and peak center are listed in the table. The units of area integration and maximum height of each peak are arbitrary. The same analysis is applied to the 28 STS curves to extract the relationship between peak area and distance from the heterointerface boundary shown in Figure 4.

For the convenience of the fitting, the peak areas in **Figure S11** are rescaled to the range of [0, 10] by dividing the appropriate power of 10.

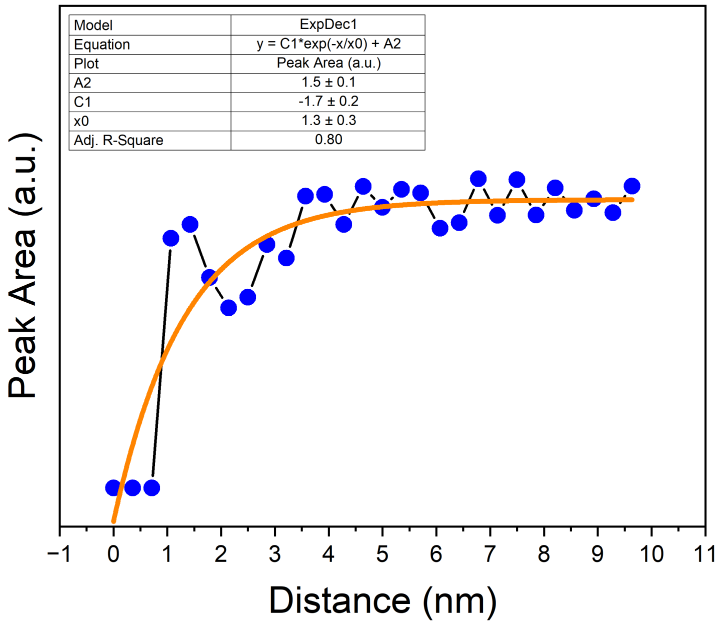

Model | ExpDec1
Equation | y = C1*exp(-x/x0) + A2
Plot | Peak Area (a.u.)
A2 | 1.5 ± 0.1
C1 | -1.7 ± 0.2
x0 | 1.3 ± 0.3
Adj. R-Square | 0.80
Peak Area (a.u.)
Distance (nm)
−1 0 1 2 3 4 5 6 7 8 9 10 11

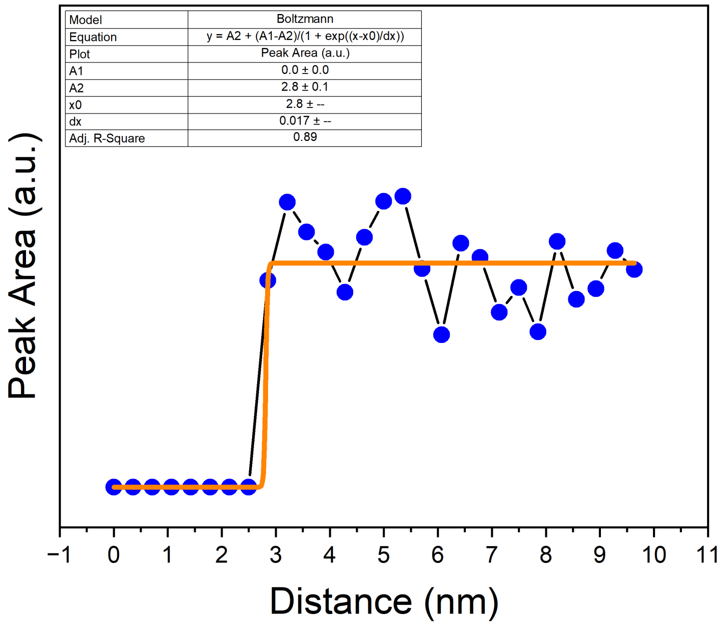

Model | Boltzmann
Equation | y = A2 + (A1-A2)/(1 + exp((x-x0)/dx))
Plot | Peak Area (a.u.)
A1 | 0.0 ± 0.0
A2 | 2.8 ± 0.1
x0 | 2.8 ± --
dx | 0.017 ± --
Adj. R-Square | 0.89
Peak Area (a.u.)
Distance (nm)
−1 0 1 2 3 4 5 6 7 8 9 10 11

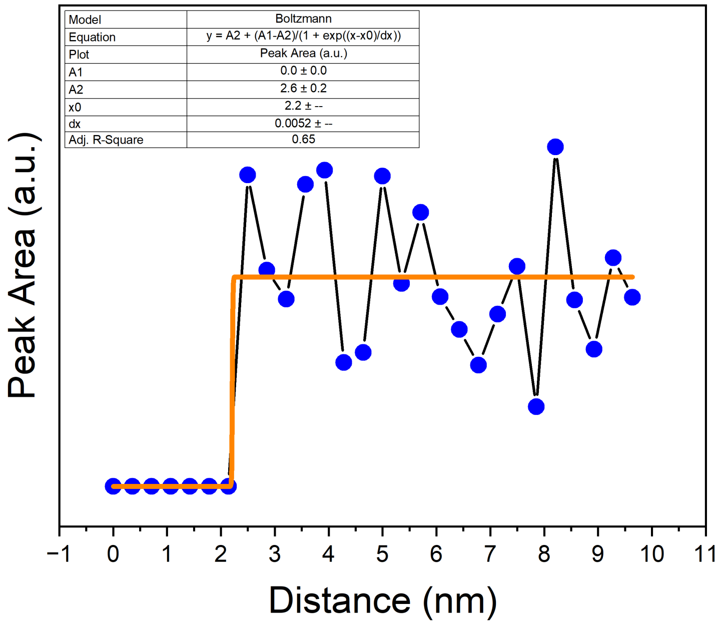

Figure S11. Detailed fitting parameters (insets) of the plots in Figure 4d-f of the manuscript. The evolution of peak area (blue dots) vs. distance from the heterointerface boundary is fitted (orange lines) by an exponential function for the density of states feature located at ~ -0.2 eV (top), and by Boltzmann functions for the features located at ~ -0.05 eV (middle) and 0.1 eV (bottom). The fitting parameter x0 is correlated to the "coupling" length of the states with the heterointerface. C1, A1, and A2 are with arbitrary unit, and x0 and dx are in nanometers.

The different line shapes are discriminated from the response of the density of state features near the boundary. In the Boltzmann function, the fitting parameters A1 and A2 correspond to the y values of the bottom and top plateaus, x0 is at the center of the transition between A1 and A2, and dx is inversely proportional to the rate of the transition, i.e, the smaller dx results in a larger slope/rate for the transition. For instance, for the feature located at 0.1 eV (the bottom graph in **Figure S11**), the peak area is negligible (A1=0) within a distance of ~ 2.2 nm from the boundary. Further away from the boundary, the peak area fluctuates around the mean, A2, which is 2.6 a.u., where a.u. stands for arbitrary unit. The transition between the two "plateaus" of A1 and A2 is relatively sharp, corresponding to a small dx. Therefore, this peak area vs. distance curve is better fitted by the Boltzmann function instead of the exponential function since the former is more suitable for fitting a "step-like" curve. For the density of states feature located at -0.2eV (the top graph in **Figure S11**), the peak area vs. distance curve is less of a "step-like" shape. Overall, the peak area increases with distance till reaching a saturation. So, this curve is better fitted by an exponential function.

As discussed above, among the four fitting parameters in the Boltzmann function, A1 and A2 are determined from the experimental data, and x0 and dx are used to describe the transition between A1 and A2. Since there are few data points along the line of transition, x0 and dx are mutually dependent on each other, i.e., the variation of one parameter could impact the fitted value of the other. To build the confidence of the fitting, the dependence of x0 on dx is analysed in **Figure S12**. We use the peak feature located at 0.1 eV as the example, which corresponds to the bottom graph in **Figure S11**. Note that the blue dots are experimental data extracted from the STS line spectra, which illustrate the evolution of peak area vs. distance from the lateral heterointerface. The orange lines are the fitting curves at the different dx values. Again, A1 and A2 are with the arbitrary unit, and x0 and dx are in nanometers.

Apparently, dx has some influence on x0, which is consistent with what we discussed earlier. Nonetheless, this influence is quite small. For dx (nm) ranging from 0.001 to 0.5, the fitted x0 (nm) value is only slightly varied between 2.1 and 2.3. Note that for dx≥0.2 the fitting curve already deviates from the overall “step-like” trend of the experimental data. It is worth noting that the quantitative analysis serves the purpose of solidifying the experimental observation. However, even without the fitting, one can still qualitatively evaluate the distance from the boundary at which the different density of state peaks emerges. This distance is correlated to the “coupling” length of the states with the lateral heterointerface, and the relevant parameter is x0 in both the Boltzmann fitting and the exponential fitting.

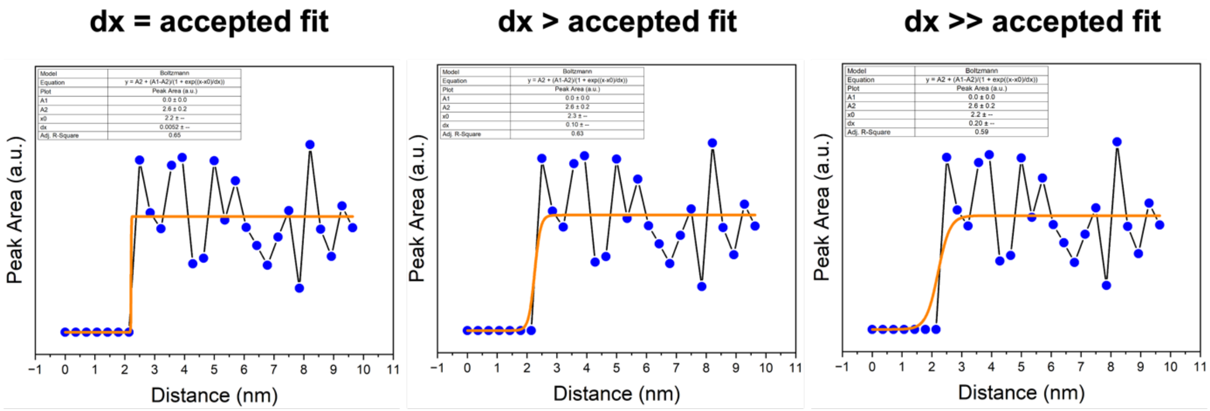

Figure S12. The influence of dx on x0. As dx are set to different values at 0.0052 (left), 0.10 (middle), and 0.20 (right), its influence on the fitted x0 value is rather limited.

Lastly, we calculate the uncertainty of x0 using the equation $\Delta x0 = \sqrt{(\Delta x0)^2_{exp} + (\Delta x0)^2_{fitting}}$. Experimentally, the accuracy of the "coupling" length is determined by the spacing between the data points of the line STS spectra. We took 28 data points across ~ 9.5 nm over the lateral heterointerface, which corresponds to a spacing of ~ 3.4 Å between two adjacent data points. Thus, $(\Delta x0)_{exp} = 0.3\ nm$. Note that this value is less than the lattice constant (~5.3 Å) of the Kagome-terminated surface. For the Boltzmann fitting of the density of states feature located at 0.1 eV, we estimate $(\Delta x0)_{fitting}$ to be about 0.1 nm based on the dependence of x0 on dx. Therefore, the overall uncertainty of x0 is ~ 0.3 nm. The calculation for the density of states feature located at -0.05 eV yields the same result. For the exponential fitting of the feature located at -0.2 eV, $(\Delta x0)_{fitting} = 0.3\ nm$, thus the overall uncertainty of x0 is ~ 0.4 nm. To summarize, the "coupling" lengths of the states with the lateral heterointerface are 1.3 ± 0.4 nm, 2.8 ± 0.3 nm, and 2.2 ± 0.3 nm for the features located at -0.2 eV, -0.05 eV, and 0.1 eV, respectively.

Section SVI: Homo-interfaces formed between the Kagome-Kagome or honeycomb-honeycomb lattices

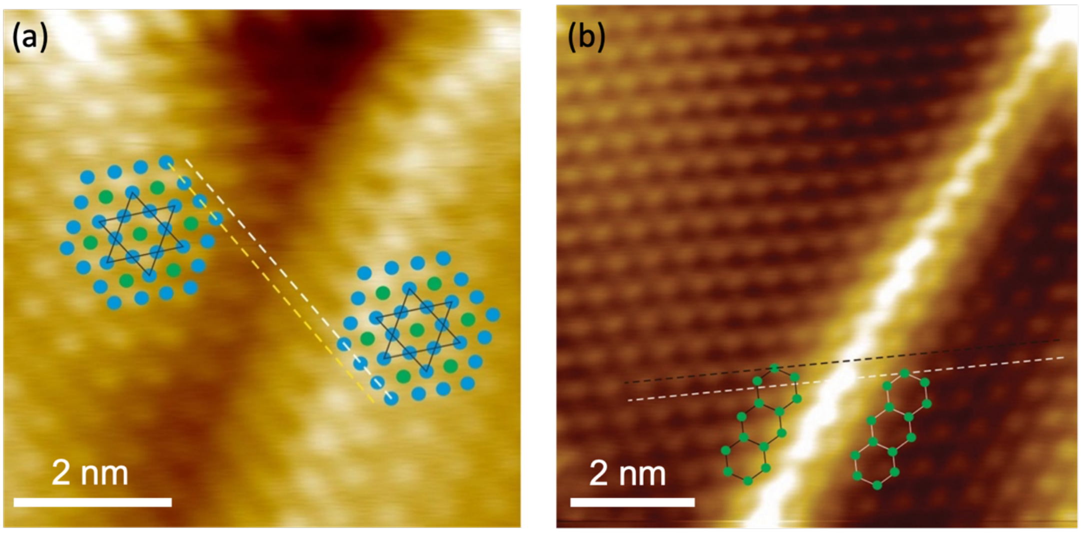

Figure S13. (a) STM image (V_s = -0.05 V, I_t = 500 pA) showing the homo-interface formed between the two different Fe_3Sn domains. The yellow and white dashed lines in (a) mark the phase slip between these Fe_3Sn Kagome domains. (b) STM image (V_s = -0.05 V, I_t = 600 pA) showing the homo-interface formed between the two Sn_2 domains. The black and white dashed lines in (b) mark the phase slip between these Sn_2 domains. The green and blue dots in (a) and (b) represent Sn and Fe atoms, respectively.

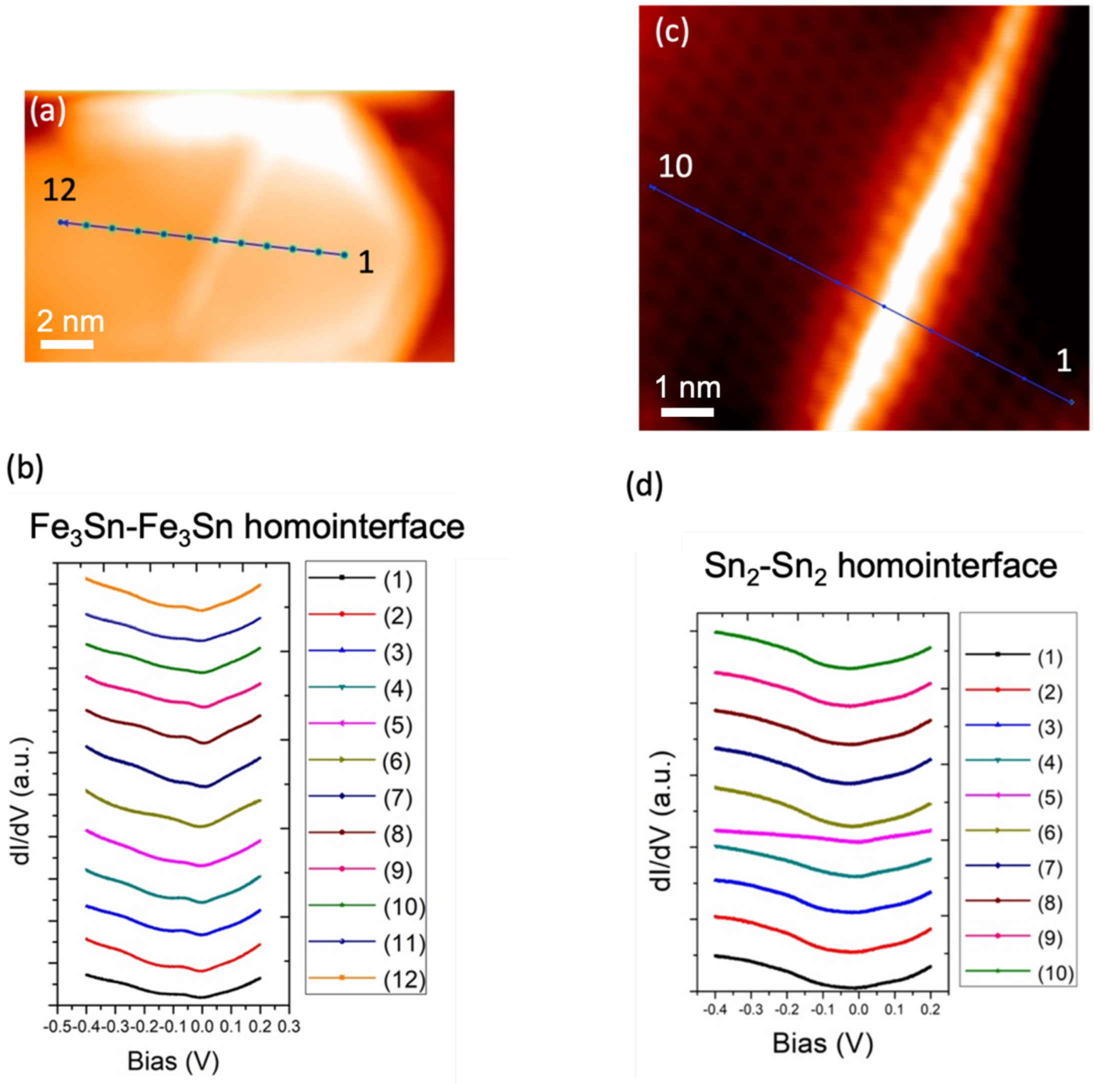

Figure S14. (a) and (b) STM image (V_s = -0.05 V, I_t = 600 pA) and the line STS spectra (setpoint: V_s = 0.4 V, I_t = 280 pA) across the Kagome-Kagome homo-interface showing consistently the electronic structures of the Fe_3Sn domain. (c) and (d) STM image (V_s = -0.05 V, I_t = 600 pA) and the line STS spectra (setpoint: V_s = 0.4 V, I_t = 280 pA) across the honeycomb-honeycomb homo-interface showing consistently the electronic structures of the Sn_2 domain. The long-range boundary effects observed in the Kagome-honeycomb heterostructures are absent here.
